# Overview on the General Approaches to Improve Gluten-Free Pasta and Bread

**DOI:** 10.3390/foods5040087

**Published:** 2016-12-09

**Authors:** Lucia Padalino, Amalia Conte, Matteo Alessandro Del Nobile

**Affiliations:** University of Foggia, Services Center of Applied Research—Via Napoli, Foggia 25 71122, Italy; lucia.padalino@unifg.it (L.P.); amalia.conte@unifg.it (A.C.)

**Keywords:** pasta, bread, gluten-free food, hydrocolloids, proteins, enzymes, starch, extrusion-cooking, starch gelatinization

## Abstract

The use of gluten-free products is increasing since a growing number of people are suffering from celiac disease and thereby need gluten-free diet. Gluten is responsible for the visco-elastic characteristics of wheat-based products; therefore, its lack makes the gluten-free products not similar to wheat-based product, with scarce textural properties. This reason constitutes the major industrial limitation. Thus, obtaining good-quality gluten-free products represents a technological challenge. This review reports the main strategies adopted to produce high quality gluten-free pasta and bread. They are mainly obtained by the utilization of specific ingredients (hydrocolloids, proteins or enzymes) to be incorporated into the standard formulation or the adoption of proper technological variables that can enhance above all the functional properties, the texture and the taste.

## 1. Introduction

The gluten intolerance disease is called celiac disease [1]. Gluten consists of two proteins called prolamines and glutenins. Among cereals, proteins of the prolamines fraction have certain names: gliadins (in wheat), hordeins (in barley), secalins (in rye) or avenins (in oat). Ingestion of these proteins leads to the inflammation, atrophy, and hyperplasia of the small-intestinal crypts of the celiac patient due to the presence of multiple proline and glutamine residues, making them resistant to gastrointestinal digestion and most exposed to deamination by tissue transglutaminase [2]. Recently, a new gluten-related syndrome, known as non-celiac gluten sensitivity (NCGS), has been identified and confirmed, through double-blind placebo-controlled trials [3,4,5]. The molecular mechanisms of NCGS have not been fully understood, although it is widely accepted that the innate immune system plays a key role in the onset of NCGS [5,6]. In recent years this syndrome has grown rapidly in adults [7,8] and in children. At present, the absence of gluten from the diet is the only way to prevent its symptoms and thus the need for high-quality products without gluten greatly contributed to develop gluten-free products. The degree of difficulty in producing gluten-free food is due to the technological/functional role of gluten in the food system. In fact, gluten is the main protein in wheat flour responsible for the visco-elastic characteristics of dough. Specifically, the wheat gluten proteins (gliadins and glutenins), in the presence of water and under mechanical work, form a continuous phase named gluten network. The flours that not containing gluten forming proteins (e.g., maize, rice, millet, sorghum) often cannot meet the requirements for bakery and pasta food processing due to the fact that their proteins are not able to form a network, but the visco-elastic quality of these flours solely depends on the properties of the starch component. The most challenging products to formulate and produce are gluten-free breads, due to the fact that gluten plays a basic role in dough, needing a minimal consistency, for example to be sheeted and/or in dough which should retain fermentation gases, such as bread, pizza, cookies, among other products. Gluten network is also necessary for pasta production. Today, most gluten-free products available on the market show poor sensory and cooking quality when compared to product based on the wheat flour [9,10]. In order to obtain good quality gluten-free products from alternative flours it is necessary to adopt balanced formulations and adequate technological processes, because of changes in the rheological properties caused by the lack of gluten. [11,12]. Processing adjustments or new ingredients (hydrocolloids, protein, enzymes) are suggested as necessary [13,14]. The common ingredients in gluten-free pasta are flour and/or starch from corn, rice, potato (or other tubers), with the addition of proteins, gums, and emulsifiers that may partially act as substitutes of gluten. The diversity of gluten-free raw materials help to rise the quantity and the quality of products for celiac people. Formulating gluten-free products requires, firstly, a thorough knowledge of the component properties of gluten-free flours and starches. Then, appropriate additives may be selected to promote a cohesive mass in the product such as pasta [15]. Several studies have focused on the investigation of alternative ingredients, including hydrocolloids, proteins and enzyme to mimic the functionality of gluten. In fact, dough enriched with these ingredients show a good aptitude to be processed to produce pasta or bread. More recently, innovative processing techniques such as high hydrostatic pressure [16] and extrusion technology [17] have been investigated as promising alternative techniques to improve rheological gluten-free characteristics. All these technologies provide a non-conventional solution for developing gluten-free foods.

This review reports the various approaches for the production of gluten-free pasta and bread, based on the utilization of new formulation made up of various ingredients (flours, hydrocolloids, proteins or enzymes) or technological options that are aimed to improve functional properties, texture and acceptability of final products. Both bakery products and pasta are examined in the following paragraphs.

## 2. Gluten-Free Flours

The raw materials used as substitutes of wheat in the celiac product formulations are maize, rice, pseudo-cereals, sorghum as well as their starches [18,19,20]. The non-conventional flours often do not have a similar quality, such as durum wheat semolina for pasta products, thus requesting proper technology to be processed. Moreover, it has been observed that gluten-free products lack dietary fibers and other important nutriments, such as certain minerals and vitamins [21], because they are usually obtained from refined flour and/or starches that are not generally enriched or fortified [22,23,24]. Nowadays, rich-protein and dietary fiber ingredients, such as pulse and vegetable flours, in combination with conventional gluten-free flours and starches can be added to increase variety and improve the nutritional quality of gluten free products [25,26].

### 2.1. Gluten-Free Flours for Pasta-Making

Maize flour is among the preferred ingredients in the preparation of gluten-free products [27,28]. Even though corn flour supplies many micro- and macronutrients, amounts of some essential nutrients are inadequate. Therefore, consumption of these products contributes only small amounts of proteins, minerals and dietary fibers, thus increasing the risk of nutritional deficiencies associated with celiac disease. Padalino et al. [29] observed that pasta of maize flour enriched with 15% chickpea flour shows an increase in dietary total fibre, proteins and fats content as compared to the control sample (100% maize flour). Chickpeas (*Cicer arietinum*) are excellent source of proteins containing high levels of complex carbohydrates and unsaturated fatty acids, rich in vitamins and minerals, and relatively free from anti-nutritional factors [30,31]. On the other hand, spaghetti samples loaded with 15% chickpea flour showed poor elasticity and increased firmness. The incorporation of hydrocolloids as pectin, guar flour and agar (2%) determined a noticeable improvement of spaghetti sensory quality [29].

Rice flour is commonly used as a raw material in preparation of gluten-free products because of its bland taste, high digestibility and hypoallergenic properties [32]. The application of rice flour produces noodles with low cooking and sensory quality, because of the weak network developed by rice protein [20]. For this reason, the addition of strengthening agents and specific treatments among which fermentation, hydrothermal, and enzyme treatments have been proposed to provide necessary network for developing gluten-free products [33,34]. Most of the extruded gluten-free products found in the market include white or polished rice and corn as main ingredients, due to their abundance, low cost and high expansion capacity [12]. However, white or polished rice is low in fibre and has relatively poor technological properties. Recently, Marti et al. [35] prepared gluten-free pasta with brown rice flour that from a nutritional point of view is considered an important gain, especially for consumers suffering from celiac disease. The greater fibre content in brown rice was responsible for a weakening of the starch network and consequently for cooking loss increase. Similarly, the inclusion of fibre in the starch matrix partially reduced the extreme firmness and springiness found in pasta from milled rice flour. Conversely, da Silva et al. [36] observed that corn flour and brown rice flour 60:40 processed via extrusion cooking recorded better sensory evaluation compared to other pasta formulations. In particular, the high shear stress and great extrusion temperature (120 °C) seemed to favour the formation of a strengthened starchy network involving the majority of starch macromolecules with a positive effect on texture of cooked pasta based on corn and brown rice flour [15].

Among cereals without gluten, sorghum represents a staple food for more than half a billion people in at least thirty countries [37]. However, despite its consumption is expanding worldwide, the sorghum crop has not yet reached its productive potential. As it is devoid of gluten, sorghum is important in human nutrition. Thus, sorghum flour may replaced for wheat flour in gluten-free products such as cakes, breakfast cereals, bread, biscuits and pasta [38]. Ferreira et al. [39] found that spaghetti with sorghum, rice and potato flours (40:20:40) reported the best cooking quality due to good density, yield, weight increase and low cooking loss. Amaranth, quinoa, and buckwheat have become increasingly popular because they improve the nutritional quality of gluten-free products, in terms of fibre, vitamins, minerals, and other bioactive components (polyphenols, phytosterols, etc.) [40]. The use of quinoa (*Chenopodium quinoa* Willd.) and amaranth (*Amaranthus* spp.) improved nutritional feature because of their good quality protein, dietary fibre and lipids rich in unsaturated fatty acids. They also contain adequate levels of minerals, vitamins, and significant amounts of other bioactive components such as saponins, phytosterols, squalene, fagopyritols and polyphenols [41]. For example, the application of quinoa and corn mixtures in the production of gluten-free spaghetti was studied by Caperuto et al. [42]. The spaghetti had reasonable physical properties in comparison to soft wheat spaghetti. Recently, several studies showed that the application of amaranth grain improves the texture and the cooking quality of pasta [18]. Moreover, compared to other gluten-free sources, macronutrients content in amaranth flour is 2–3 times higher and it is similar to wheat [25]. Incorporation of amaranth flour in rice pasta combined with extrusion-cooking improves textural and nutritional quality of final product. Addition of 25% amaranth flour significantly improves the nutritional characteristics of rice-based pasta without noticeable decrease of cooking quality [43]. For example, the amaranth shows a higher dietary fiber and mineral content and the proteins have better amino acid nutritional balance with respect to other vegetable flour [44]. Amaranth flour has already been used to enrich cereal-based foods, including gluten-free pasta. Cabrera-Chávez et al. [44] observed that the addition of amaranth flour in pasta based on rice flour combined with extrusion-cooking improves the textural and nutritional quality of the final product. Specifically, the best results in terms of textural quality of pasta are obtained when starch in rice flour is allowed to interact during gelatinization with amaranth proteins that are simultaneously undergoing thermal denaturation in the extrusion-cooking process. Florida et al. [45], reported that the vermicelli-type pasta obtained using pregelatinised cassava starch and bagasse (70:30) flour, cassava starch and amaranth flour in the proportion of 10:60:30, show the best results in the cooking quality tests, such as a lower solids loss to the cooking water when compared the whole wheat pasta sample.

Recent studies evaluated the potential use of resistant starch (RS) as a fibre-enriching ingredient in gluten-free pasta. Specifically, Foschia et al. 2016 [46] reported also that the addition of RS improved the quality of gluten-free pasta owing to its ability to increase the firmness and decrease the cooking loss and stickiness of cooked pasta.

It has been recently reported that the intake of refined sugars in celiac patients is high [47], since the addition of good source of indigestible carbohydrates is important. Several studies suggested that consumption of unripe plantain (*Musa paradisiaca* L.) exerts a beneficial effect on human health; this is associated with indigestible components as resistant starch (RS) [48]. Flores-Silva [49] observed that gluten-free spaghetti made from a mixture of unripe plantain, chickpea and maize flours showed to have great potential for commercial application due to firmness, hardness, cohesiveness and chewiness characteristics, similar or higher than semolina wheat spaghetti.

### 2.2. Gluten-Free Flours For Bread-Making

Rice flour is the most commonly used in gluten-free bread formulations due to the fact that it is widely available and inexpensive. It is white in color, bland in taste, easily digested, and hypoallergenic. Although these advantages, rice flour presents technological limitations in bread-making due to the poor functional properties of its proteins [50,51]. Therefore, different additives like hydrocolloids, proteins, enzymes, and emulsifiers have been used to improve volume, texture, appearance, acceptance, and shelf life of rice breads [52]. Rice-based ingredients, such as brown rice flour [53], defatted rice bran [51], or extruded rice flour [54] are as well used.

Increasing number of studies has investigated the application of pseudo-cereals in the production of gluten-free bread. The nutritional properties and baking characteristics of amaranth, quinoa and buckwheat have been assessed in gluten-free matrices [40], achieving breads with superior nutritional features and acceptable degree of linking. Gambus et al. [55] reported that the feasibility of amaranth as an alternative gluten-free ingredient to improve the nutritional quality of gluten-free breads. In a more recent study [56], amaranth-based gluten-free bread fortified with iron was successfully formulated. Renzetti et al. studied the application of buckwheat as a composite flour in the development of high-quality gluten-free breads [53]. In other recent studies, both nutritional properties and baking characteristics of quinoa, amaranth and buckwheat have been assessed [40]. The authors found that the replacement of potato starch with a pseudo-cereal flour improves the nutritional attributes of bread due to the increase in proteins, fibers, polyphenol compounds content and high antioxidant activity. As compared to the control, buckwheat and quinoa bread recorded a higher volumes and a softer crumb structure [57].

Fruit flours have also good prospective as raw materials in baked product manufacture. For examplechestnut flour may be used in gluten-free flour bread because of its nutritional and health benefits. Chestnut flour contains great quality proteins with essential amino acids (4%–7%), relatively high amount of sugar (20%–32%), starch (50%–60%), dietary fiber (4%–10%), and low amount of fat (2%–4%). It also contains vitamin E, vitamin B group, potassium, phosphorous, and magnesium [58]. The baked products based on chestnut flour have good nutritional quality but poor sensory quality because of low volume and unacceptable dark color and undesirable hardness. These defects may be caused by the inadequate starch gelatinization, high amount of sugar and fiber. Demirkesen et al. [59] found that when bread was prepared using only chestnut flour, hard structure with the low volume were observed because of the rigid and compact structure of the fibrous chestnut flour dough. The rise in the chestnut flour content declined the loaf volume but rose the hardness of bread. Relatively high sugar content of chestnut flour may also hinder or decline the starch gelatinization during baking, leading to small specific volume and firm texture of bread. Sugars are known to delay starch gelatinization by reducing the water activity of the system and stabilizing the amorphous regions of the starch granule by interacting with starch chains [60]. Thus, breads cannot entrap the gas bubbles leading to lower volume and harder structure. As compared to bread made with chestnut/rice flour ratio 30/70, bread produced by using only chestnut flour had lower flavor score [60]. This may be due to the off-flavor formation as result of the Maillard reaction. In addition, the sugar of the chestnut flour triggered Maillard and caramelization reactions, resulting in an undesirable dark color. Formulations based on a single starch or on starch mixture, with or without flour, have been developed using additives (hydrocolloids, protein) added for nutritional purposes to improve bread [61]. In particular, different application have been studied such as the use of native, gelatinized [62], or modified starches [63] in order to make better gluten free quality Moreover, starch blends (potato, corn, rice, and tapioca) have been used in several gluten free bakery products improving the volume, texture, color, crumb grain, flavor, and mouthfell properties of wheat-based items [64]. Onyango et al. [61], found that the combination of 50% sorghum flour and 50% cassava starch results in breads with better crumb properties and texture as compared to the combinations of sorghum flour with 10% to 40% cassava starch or 10% to 50% corn, rice, or potato starches. Most probably cassava starch produces a hard elastic mass during gelatinization, yielding a higher cohesion with respect to the starches of other cereals and tubers and entrapping air bubbles in the dough due to its rubbery and sticky properties. This allow you to the formation of flexible gas cells that hold carbon dioxide during proofing and baking, thus improving bread volume and texture [62].

## 3. Hydrocolloids Adoption in Gluten-Free Products

Food hydrocolloids from seaweed, plants, microorganisms, as well as modified bio-polymers created by enzymatic or chemical modification of cellulose and starch, are compounds with a great molecular mass, containing a hydrophilic string, often with colloidal properties (ability to bind large amounts of water firmly, up to one hundred times its mass and thus extending product shelf life). These compounds are applied as ingredients in the food industry to improve texture and taste and to prolong the shelf life, mainly of bread [65,66]. The application of hydrocolloids in the gluten-free sector depends on their ability to raise the water-holding capacity, viscosity, hydration rate and the effect of temperature on hydration because for most hydrocolloids, viscosity decreases with increasing the temperature [67].

### 3.1. Hydrocolloids Adoption in Gluten-Free Pasta

Hydrocolloids incorporation can be an easy solution for bettering pasta-cooking quality. As compared with semolina pasta, one prepared only from gluten-free flour is generally considered to be of low quality; it does not tolerate over-cooking, it is sticky, and, above all, it is characterized by relevant cooking loss. Hydrocolloids or gums improve firmness, give body and mouth-feel to gluten-free pasta because of their ability to make a gel in little quantity that provide high consistency at room temperature. Padalino et al. [11] reported that hydrocolloids such as chitosan and carboxymethylcellulose improved sensory properties (elasticity, adhesiveness and bulkiness) of maize pasta with oat bran because hydrocolloids help to gelatinize maize starch to form a stable network that improves pasta structure. In particular, Padalino et al. [11] reported that chitosan and carboxymethylcellulose decrease the adhesiveness and bulkiness of spaghetti samples based on maize enriched with oat bran because of chemical groups of hydrocolloids that are capable to form a stable polymeric network that entraps starch granules, slowing down the amylose release. Several authors reported the effect of hydrocolloids on starch gelatinization and found that some hydrocolloids are capable of limiting the gelatinization of starch granules due to their high hydrophilic nature. In other words, hydrocolloids compete with starch for water uptake thus modifying the gelatinization process [68,69,70,71]. They also suggested that chitosan had a higher affinity for pre-gelatinized starch, developing a more continuous starch network and promoting greater viscosity of spaghetti dough. Silva et al. [72] found that hydrocolloids with a high water binding capacity such as hydroxypropylmethylcellulose and xanthan gum, could diminish the gelatinization starch particles due to the fact that they compete for the available water. Padalino et al. [30] reported that the addition of pectin in spaghetti of maize flour enriched with chickpea flour recorded the lowest gelatinization degree as compared to control samples or other samples with guar gum and agar. Moreover, Padalino et al. [11] observed that the addition of hydrocolloids causes difference in the rheological properties of starch network (gum gellan gum, carboxymethylcellulose, pectin, agar, egg protein powder, tapioca starch, guar seed flour and chitosan) mainly due to changes in granule gelatinization, gum solubilization, or starch–gum interactions. Raina et al. [73] also observed that the addition of guar gum in pasta based on the rice flour resulted in a rise of hardness. Table 1 shows some hydrocolloids used in gluten free pasta-making.

### 3.2. Hydrocolloids Adoption in Gluten-Free Bread

Different hydrocolloids have also been applied to gluten-free bread formulation [85,86,87]. When hydrocolloids interact with water during the bread-making process, they produce a gel network that serves to rise dough viscosity and strengthen the boundaries of expanding cells, increasing gas retention capability during proofing and baking, improving bread volume, structure, texture and appearance [88,89,90]. Hydrocolloids also show a “water-release” effect necessary for starch gelatinization during baking [29]. Eduardo [91] found that the incorporation of carboxymethylcellulose to composite cassava-maize-wheat bread suave improved bread quality parameters such as specific volume, crust color, and crumb texture. Similar effects on bread specific volume have been observed when adding hydroxy-propyl-methyl-cellulose to gluten-free maize-teff bread [92] and pectin to gluten-free formulations [87]. These findings might be a result of the formation of a gel network during oven heating that strengthens the expanding cells of the dough and, as a result, improves gas retention and bread volume [93].

Loaf volume rose up to a certain xanthan gum supplementation level, but further increase in polymer concentration resulted in volume decrease [88]. It seems that with xanthan gum addition at high concentrations, dough exhibits too high resistance and consistency, which cause a limited gas cell expansion during proofing [87]. Similarly, Haque et al. [94] observed no influence of xanthan incorporation into a rice flour bread, whereas Schober et al. [95] found a decrease in loaf volume of gluten-free breads from sorghum with increasing xanthan gum levels. Moreover, the introduction of xanthan gum into the mixture of hydrocolloids used as a gluten replacement significantly influenced hardness of gluten-free bread and to the lesser extent their cohesiveness, due to the different interactions between starch and hydrocolloids [96]. It is of interest to observe that the addition of xanthan gum increased batter consistency and improved gluten-free bread quality (100% rice flour), which led to bread with high volume, increased cell average size and lower crumb firmness and staling rate over storage; similarly, it improved bread overall appearance with respect to the other hydrocolloids [96].

Furthermore, hydrocolloids could be the easiest way to rise the content of dietary fibre in gluten-free bakery products. In general, the gluten-free products are characterised by much smaller nutritional value, due to the fact that they lack many important nutrients, such as vitamins, proteins, minerals and dietary fibre. One of these ingredients used by the food industry, classed as a dietary fiber, is β-glucan, a non-starch polysaccharide that it is located in the endosperm cell walls of oat constituting about 75% of endosperm cell walls [11]. Lazaridou et al. [87] use β-glucans into gluten-free formulations based on rice flour, corn starch, and sodium caseinate and examine their effects on bread sensorial properties. Specifically, the addition of β-glucans resulted in increased bread loaf volume at both studied levels (1% and 2%), crumb porosity at 1% level, and significantly increased crumb firmness when added at 2% concentration [87]. Table 2 shows some hydrocolloids used in gluten free bread-making.

## 4. Proteins and Enzymes Adoption in Gluten-Free Products

The application of proteins as structuring ingredients is an interesting alternative for production of gluten-free products [109]. These ingredients could be incorporated in various forms, as constituents of gluten-free flours (e.g., rice, soy, pea) or in the form of concentrates and isolates [114]. Addition of non-gluten proteins in the production of gluten-free products positively affects structure, texture, sensory properties as well as nutritional quality in particular, reduces amino acid deficit. Other additives successfully applied in several gluten-free foods are the enzymes for their unique ability to modify proteins functionality and promote proteins cross-linking [54,115,116,117,118]. The addition of enzymes in gluten-free dough improves rheological characteristics, handling properties and shelf-life. Enzymes such as glucose oxidase and transglutaminase and many other enzymes, are currently used in gluten-free applications [118,119,120].

### 4.1. Proteins and Enzymes in Gluten-Free Pasta

In gluten-free pasta great incorporation of albumen improves texture properties [13]. The addition of egg proteins has a positive effect on the cooking quality, resulting in firm and elastic pasta with poor cooking loss [74]. Recent studies observed an improvement in pasta texture when milk and egg proteins were added in the formulation [75,76,77]. The application of high egg-proteins in gluten-free pasta made from parboiled rice flour improves the cooking quality because the proteins may form a more compact network that does not allow for any release of organic matter during cooking. Marti et al. [121] found that as compared to the whey proteins, the egg albumen was significantly more efficient to decrease cooking loss. Recent studies report that the addition of emulsifiers (1.2%) and egg white powder (6%) improved the texture (impart firmness) and cooking quality (low cooking loss) of gluten-free pasta based on quinoa, amaranth and buckwheat flour blends [13].

Recently, the effects of dairy proteins on both rheological and mechanical properties of fresh hand-made tagliatelle obtained from pseudo-cereal flours were investigated [122]. Moreover, dairy proteins incorporation to sweet potato produce high-quality pasta and low starch digestibility due to strong starch–protein network formation [123,101]. Proteins such as collagen and lupine proteins were also tested in the production of gluten-free products [124]. The combination of amaranth, quinoa, and buckwheat (40:40:60) with 6% of egg white powder and 1.2% of emulsifier improved cooking quality of gluten-free pasta [118]. TG (protein-glutamine c-glutamyl transferase, EC 2.3.2.13) is able of catalysing the training of non-disulphide covalent crosslinks between ε-amino groups of lysine residues and peptide-bound glutaminyl residues in proteins such as cereal protein (wheat, barley and rice) [74,125]. In fact, Yalcin [78] found that the addition of the TG improved machining ability of dough based on rice flour, caused smooth noodle surface and improved also rice noodle quality in terms of total organic matter values. Sibakov [79] observed that the enzyme addition in the pasta with faba bean flour mainly acted on the properties of pasta reducing the in vitro starch digestibility and rose some textural parameters (cohesiveness, chewiness, resilience and hardness), likely because of crosslinks in the protein network, with respect to semolina pasta. Table 1 shows some protein and enzymes used in gluten free pasta-making.

### 4.2. Proteins and Enzymes in Gluten-Free Bread

Egg proteins addition in gluten-free bread formulation is able to form cohesive films essential for stable foaming and gas retention during baking, and help to build structure in bread product [97].

In addition, the incorporation of dairy proteins to gluten-free bread formulations improves texture and rise volume, taste and crust color of product. Crust darkening is based on the caramelization and Maillard reactions, due to the milk components including protein and lactose [17]. In addition, the diary proteins as caseins positively influence nutritional value (good sources of calcium and amino acids such as lysine, methionine and tryptophan) and quality of gluten-free products [98,126]. The addition of casein and egg white to dough based on rice flour improved handling and processing [121]. Other formulations include isolates and concentrates of dairy proteins characterized by great water binding capacity and ability to form gel-like structures [115]. However, the application of dairy proteins is limited because dairy proteins themselves can be allergens and frequently celiac disease is also accompanied by lactose intolerance.

The cereal proteins corn and kaffirin have been also applied to gluten-free bread [99,115,127]. Andersson et al. [128] observed that the addition of corn protein with hydrocolloids noticeably influences dough rheology, improves bread structure and enhances its volume. Schober et al. [100] also reported a positive influence of corn on visco-elastic properties of dough, good gas retention and rose bread volume. Another group of proteins applicable to gluten-free products is derived from the legume seeds. This addition improved nutritionally valuable, due to high lysine content, which is a limiting amino acid in cereal products [129]. Isolates and concentrates of these proteins are produced in great quantities, in particular from soy and pea. Marco and Rosell [102] observed that the addition of legume proteins cause a soar in water absorption and modify textural properties of bread. In other studies a rise of specific volume and improvement of sensory quality were observed after pea protein supplementation, which was also accompanied by a decreased retrogradation [89]. The presence of lupine and pea preparations improved sensory parameters, providing more acceptable color and smell with respect to the control sample, whereas soy caused a decline of all sensory attributes (i.e., low volume) [63]. The addition of proteins generally causes a rise in bread hardness and in enthalpy of retrograded amylopectin, during bread storage.

Cyclodextrin glycosyl transferase (CGTase) [103] as well as amylases, have been employed to increase shelf-life in gluten-free breads [130] because both enzymes hydrolyze starch and reduce its retrogradation. Similarly, the use of lipases, which produce emulsifiers, also reduces this phenomenon [131] since emulsifiers also present anti-staling properties. Furthermore, in bread-making industry amylases from different origins (cereal, fungal and microbial) are in use to increase bread volume, improve crust and crumb color and develop flavor [132].

Rheological and handling properties of oat dough have been enhanced by the incorporation of TG and exogenous proteins due to protein cross-linking action [104]. The addition of TG to rice dough influenced both rheological properties and consistency [75,105]. The cross-linking of rice proteins by the incorporation of TG was confirmed by the decline of free amine groups [106,118,121]. The increased water retention with growing TG also highlights the structural modification of proteins, allowing more water to bind. The addition of TG along with non-gluten proteins can notably recuperate the network structure of gluten-free batter [133].

The incorporation of strengthening agents as enzymes have been proposed to provide necessary network for processing rice products [18,33,34]. Among them, cross-linking of rice protein using TG improved the functionality of rice proteins [74,134]. TG catalyses the reaction between an ε-amino group on protein bound lysine residues and the c-carboxyamide group on protein-bound glutamine residues, leading to the covalent crosslinking of the proteins [133]. TG treatment improved the consistency and the elasticity of brown rice batters and determined noticeable improvement of textural quality of bread [118]. Instead, gluten-free products made of no-gluten cereals have very small proteins content and lysine residues which TG probably uses to make crosslinks. Because of this, the reinforcement of rice products with protein is inevitable, and proteins from various sources, such as pea, soybean, whey and egg albumin, have been used to rise the reactivity of protein with TG and to improve the nutritional value of gluten-free products [134].

Protein polymerization can also get better bread-making performance of gluten-free flours by improving elastic-like behavior of dough [118]. Glucose oxidase (GO) treatment has shown to enhance the elastic-like properties of sorghum and corn flour. This result of aggregated protein structure enhanced the continuity of the protein phase in the mixture of sorghum and corn flour [115,107]. In addition, high levels of free sulfhydryl groups in these flours favored protein polymerization by disulphide bonds, resulting in the aggregates. Yano et al. [107,108] recently reported that incorporation of glutathione, a tri-peptide, to rice dough increases the loaf volume of bread due to the fact that glutathione works by disrupting disulfide linked structures present in the grain that are responsible for resistance to dough deformation. Protein hydrolysis by bacterial proteases improves bread quality [135]. Renzetti et al. [118] also reported that a bacterial protease could improve the quality of gluten-free brown rice bread, where the loaf volume increases and both crumb hardness and chewiness decrease. The treatment by commercial protease from *Bacillus stearo thermophilus* (thermoase) improves bread quality, i.e., good crumb appearance, high volume and soft texture, depending on the amount of added enzyme. Table 2 shows some proteins and enzymes used in gluten free bread-making.

## 5. Technological Options to Improve Gluten-Free Products

The most common approach to enhance gluten-free bread and pasta quality is to modify starch functionality and its macromolecular structure. Starch plays a key role in gluten-free food production due to the fact that it is the main ingredient of gluten-free raw materials. Gluten-free food technology primarily relies on dough heating and cooling operations that exploit two phenomena: before starch gelatinization and, subsequently, its retrogradation. Specific aspects were discussed in the following two paragraphs dealing with gluten-free pasta and bread.

### 5.1. Technological Options to Improve Gluten-Free Pasta

The first technological approach used for production of gluten-free pasta is focused on the use of heat-treated flours, where starch is gelatinized [15]. The heat-treated flour can be transformed into pasta through the continuous extrusion press also used in durum wheat semolina pasta-making. However, one of the most suitable technologies for gluten-free pasta-making is extrusion-cooking process in which native flour is treated with steam and extruded at high temperatures (more than 100 °C) for short time in order to promote starch gelatinization directly inside the extruder-cooker. As recently reported by Wolf [80] the crystalline starch macromolecules are converted into a more amorphous material able to produce a malleable product. As a consequence, pasta prepared by extrusion-cooking shows high firmness, texture and flavor after cooking, as compared to pasta obtained by a conventional extruder [81]. Extrusion-cooking process has been successfully applied for production of pasta based on corn flour [12,81,136]. The gluten-free flour was before heat-treated in the extruder for contact with a heated wall and/or steam injection, and subsequently, extruded, formed, shaped and lastly dried: the pasta shows an improvement of the cooking quality in terms of low cooking loss [12,135]. Both these processes have been applied [137] to the production of pasta based on native rice flour. Since a continuous and regular protein network was lacking, starch polymers were less trapped in the rice matrix, resulting in a product with great cooking losses (10 g/100 g), two-three times higher with respect to the durum wheat semolina pasta.

In addition, physical treatments such as annealing have been often applied to starch to change the native physicochemical properties in order to meet different industrial requirements [138]. Specifically, the annealing consists in the treatment of starch with much of water (more than 40%) to lower temperature gelatinization (for rice 50–60 °C), and heat-moisture treatment (treatment at small moisture and great temperatures, 100–120 °C for rice) [139]. Both of them increase starch crystallinity, granule rigidity, and polymer chain associations [139,140,141]. These specific hydrothermal treatments inhibit granule swelling, retard gelatinization and increase starch paste stability [34,142], thus enhancing texture properties and cooking behavior of rice noodles [34,82]. The optimization of hydrothermal treatment conditions produces rice noodles with different cooking quality [143]. In particular, heat-moisture treatment is more suitable for semi-dried and dried noodles characterized by great tensile strength and gel hardness whereas annealing is appropriate for producing fresh rice noodles that require a soft texture. Although the betterment associated with the use of these treatments, the utilization of pregelatinized flour is generally considered a less expensive approach for improving rice noodle quality. In fact, Raina et al. [73] found that textural quality of both uncooked and cooked pasta improved considerably when pre-gelatinized rice flour was used.

It should be noted that drying temperature rose pasting temperature of starch and hence cooking quality of the resulting pasta [144]. Zhang et al. [83] observed that water absorption of starch declined with rising drying temperature; even the formation of lipid-amylose-complexes could contribute to rise texture firmness and reduce cooking loss. D’amico et al. [84] reported that high drying temperature and pre-drying (at lower temperature) influenced texture properties, in particular cooking loss and protein solubility decreased indicating superior structural integrity of gluten-free pasta based on amaranth/quinoa/buckwheat and millet/white bean. However, although important quality parameters were improved, the performance of wheat pasta was not yet reached. In fact, the gluten-free pasta had low elasticity and high proteins solubility with respect to wheat pasta. Table 1 shows some technological options used in gluten free pasta-making.

### 5.2. Technological Options to Improve Gluten-Free Bread

Heat treatment has been proposed to be a valid method to improve bread quality specially in weak, poor flour. Russo et al. [145] patented the heat-treated process flour using a temperature ranged between 100 °C and 115 °C for 60 min. Heat exposure denatures enzymes and proteins in the flour while growing batter expansion [145]. In the bread preparation, the use of heat-treated flours has been shown to increase viscosity, resistance and stiffness [110]. These elements lead to an increase of dough elasticity and produce positive effects on oven spring and loaf volume [111]. Flours or starch with a hydrothermal pre-treatment process during which starch is gelatinized report great thickening properties and high water absorption also at room temperature. Their effect is therefore comparable to that of hydrocolloids [33,34], even if results are still very variable and it is significant to adjust dough moisture to attain optimal rheological properties. Partial starch gelatinization of raw material has also been obtained through high hydrostatic pressure. The strategy first developed for gluten breads [112] was applied to sorghum dough in the production of sorghum breads [16]. Recently, it has been observed that pressures higher than 300 MPa gave higher sorghum dough consistency due to pressure-induced gelatinization of starch The substitution of 20 g·kg^−1^ sorghum flour with flour treated at 600 MPa decreased the staling of bread, because it is well that high pression inactivate enzymes that are mainly responsible for shortening the products shelf-life. However often the incorporation of higher amounts of pressure-treated flour decreased specific volume and bread quality due to the high starch gelatinization occurring during pressure application [112]. In addition, Cappa et al. [113] observed that bread obtained from corn starch and rice flour pre-treated at 600 MPa for 5 min at 40 °C, has lower the staling rate of bread crumb and loaf volume with respect to control sample (no treated). Table 2 shows some technological options used in gluten free bread-making.

## 6. Conclusions

Despite the considerable efforts addressed in the last few decades to produce gluten-free pasta and bread with sensory characteristics analogous to durum wheat products, in reality the products currently present on the market are yet far from what the consumer is looking for. Therefore, there is no raw materials, additives or ingredients (proteins, hydrocolloids, and enzymes) that can completely substitute the gluten, but the combination of raw materials, ingredients and proper production technologies could promote the production of gluten-free product of good quality. The results of the reviewed studies stimulate further research on the improvement and optimization of new gluten-free pasta and bread formulas. In particular, extensive research on the improvement of nutritional quality is also needed so that a gluten-free pasta or bread with both high technological and nutritional properties can be produced and made available to the celiac sufferers to improve their quality of life.

## Figures and Tables

**Table 1:** Main ingredients and technological options used in the pasta-making.

**Hydrocolloids**	**Effects**	**References**
Chitosan Carboxymethylcellulose	Decrease of the adhesiveness and bulkiness.	[11]
Hydroxyl-propyl-methyl-cellulose xanthan gum	Decrease of the gelatinization starch.	[11]
Gum gellan gum Carboxymethylcellulose Pectin Agar Egg protein powder Tapioca starch Guar seed flour Chitosan	Changes in the rheological properties of starch network.	[11]
Guar Gum	Increase of the hardness.	[72]
**Proteins and Enzymes**	**Effects**	
Milk proteins Egg proteins Whey proteins	Improvement in pasta texture and cooking quality.	[13,74,75,76,77]
Transglutaminase	Improvement of the machining ability of gluten free dough. Improvement of the cohesiveness, chewiness, resilience and hardness.	[78,79]
**Technological Options**	**Effects**	
Extrusion-cooking process	Increase of the firmness, texture and flavor after cooking. Improvement of the cooking quality in terms of low cooking loss.	[80,81]
Annealing	-	[82]
High drying temperature	Increase of the firmness. Decrease of the cooking loss. Decrease of the elasticity. Increase of the proteins solubility.	[83,84]

**Table 2:** Main ingredients and technological options used in the bread-making.

**Hydrocolloids**	**Effects**	**References**
Xanthan gum	Increase of the resistance and consistency dough. Improvement of the hardness of gluten-free bread. Improvement of the dough cohesiveness.	[87]
β-glucans	Increase of the loaf volume (1% and 2%), crumb porosity (1%) crumb firmness (2%).	[87]
Carboxymethylcellulose Hydroxy-propyl-methyl-cellulose Pectin	Improvement of the bread quality parameters: specific volume, crust color, and crumb texture.	[91,93,96]
**Proteins and Enzymes**	**Effects**	
Eggs proteins	Increase of the dough loaf volume.	[97]
Dairy proteins	Improvement of the texture loaf volume, taste and crust color.	[98]
Cereal Protein: Corn, Kaffirin	Improvement of the bread structure and loaf volume.	[99]
Legume protein: Soy, pea, lupine	Improvement of textural properties. Improvement of some sensory parameters (color, smell).	[100,101]
Cyclodextrin glycosyl transferase	Increase of the shelf-life in gluten-free bread.	[102,103]
Transglutaminase	Improvement of the rheological properties (consistency and elasticity). Improvement of the textural quality bread.	[104,105,106]
Glutathione	Increase of the loaf volume.	[107,108]
Protease	Increase of the loaf volume and crumb hardness Decrease of the bread chewiness.	[109]
**Technological options**	**Effects**	
Heat treatment flour	Increase of the dough viscosity, resistance and stiffness. Increase of the dough elasticity and loaf volume.	[110,111]
High hydrostatic pressure (600 MPa)	Decrease of the specific volume and bread quality. Decrease of the staling rate of bread crumb Decrease of the loaf volume bread.	[112,113]
